# Phylogeographic Substructuring in the Southernmost Refugium of the European Common Frog *Rana temporaria*

**DOI:** 10.3390/ani14101430

**Published:** 2024-05-10

**Authors:** Marija Ilić, Vanja Bugarski-Stanojević, Bogdan Jovanović, Gorana Stamenković, Katarina Zorić, Momir Paunović, Jelka Crnobrnja-Isailović

**Affiliations:** 1 Department of Hydroecology and Water Protection, Institute for Biological Research “Siniša Stanković”, National Institute of Republic of Serbia, University of Belgrade, 11108 Belgrade, Serbia; marija.ilic@ibiss.bg.ac.rs (M.I.); katarinas@ibiss.bg.ac.rs (K.Z.); mpaunovi@ibiss.bg.ac.rs (M.P.); 2 Department of Genetic Research, Institute for Biological Research “Siniša Stanković”, National Institute of Republic of Serbia, University of Belgrade, 11108 Belgrade, Serbia; vanjabs@ibiss.bg.ac.rs (V.B.-S.); gorana_stamenkovic@yahoo.com (G.S.); 3 Department of Evolutionary Biology, Institute for Biological Research “Siniša Stanković”, National Institute of Republic of Serbia, University of Belgrade, 11108 Belgrade, Serbia; bogdan.jovanovic@ibiss.bg.ac.rs; 4 Department of Biology and Ecology, Faculty of Sciences and Mathematics, University of Niš, 18000 Niš, Serbia

**Keywords:** *Rana temporaria*, phylogeography, *16SrRNA*, *MT-CYTB*, southernmost haplotypes, haplotype diversity, Balkan Peninsula, conservation

## Abstract

**Simple Summary:**

*Rana temporaria* is one of the most widespread Palearctic brown frogs, with two main genetic clades in Europe, geographic spatial pattern of which is insufficiently known. We analyzed samples from the understudied western and central Balkans to evaluate the haplotype diversity of widely used *16SrRNA* and *MT-CYTB* mitochondrial gene sequences and to recognize the contour of a possible contact zone between the main clades. The results revealed a suture zone between the Western and Eastern Clades in the central part of the Balkan Peninsula. Overall, haplotype diversity in the western and central Balkans sample is high. Harboring both main genetic clades of *R. temporaria* qualifies the Balkan Peninsula as another important center of species’ genetic diversity, as well as rich in unique haplotypes.

**Abstract:**

*Rana temporaria* is one of the most widespread Palearctic brown frogs. We aimed to clarify distribution pattern of two main genetic clades in the understudied Balkan peninsula by using *16SrRNA* and *MT-CYTB* sequences, already widely applied in analyses of populations from other parts of Europe, while focusing on the broad area along the Morava river (central Balkans) as a known gap in the species distribution. Additionally, we were interested in revealing the extent of haplotype diversity within the main genetic clades in the Balkans, particularly around the supposed suture zone. The results revealed a suture zone between the Western and Eastern Clades in the central part of the Balkan Peninsula. This indicated the existence of a historical barrier between the Balkan Mountain Belt and geographically close mountains surrounding the Vlasina Plateau (Rhodope/Serbian–Macedonian Massif). The overall observed haplotype diversity in populations of *R. temporaria* from the Balkan Peninsula seems high. Harboring both main genetic clades of *R. temporaria* qualifies the Balkan Peninsula as another important center of species’ genetic diversity, as well as rich in unique haplotypes. This points out the necessity of applying conservation measures focused on the common European frog populations and habitats in this part of the species’ distribution area.

## 1. Introduction

*Rana temporaria* (“the common frog” or “the European Common frog”) is one of the most widespread and abundant amphibians in Europe [1], except in the southern parts of the continent, where its distribution is apparently fragmented [2]. It spreads from northern Spain, through western, north, and central Europe, to eastern Europe and further eastward to northern Asia [3]. In the South of Europe, it also inhabits northern and central parts of Italian Peninsula and the higher mountain parts of the Balkan Peninsula [1,2,4]. Some European populations are recorded at very high elevations (up to 2800 m) in the Alps and Pyrenees [5,6,7]. The common European frog has the highest genetic variation of all western Palearctic brown frogs [8]. Although designated as “Least Concern” [3], it could be one of the most fragile anurans in southern Europe, regarding the impact of projected climate change [9,10].

European brown frogs’ lineage separated from their central Asian ancestor about 22.2 million years (Mya later in the text) ago, in the Early Miocene [11]. Radiation over Europe and speciation were later influenced by climatic oscillations between warmer and colder periods, particularly in the Pleistocene. Cold periods would initiate retreat in refugia, followed by genetic divergence as a consequence of spatiotemporal isolation, while, during warmer periods, species would expand their area. The major refugial regions for European flora and fauna during the time of Pleistocene were the three southern ones (the Iberian, Apennine, and Balkan peninsulas) and one in southwestern Russia [12]. Somewhere at the boundary between the Early and Late Pliocene, about 3 Mya, the *R. temporaria* species group split into five lineages, one of them being *R. temporaria* (including the recently proclaimed species *R. parvipalmata*) [6].

The first molecular genetic study on *R. temporaria* was undertaken by Pidancier et al. [13], using the cytochrome b mitochondrial (mt later in the text) desoxyribonucleic acid (DNA later in the text) sequence (*MT-CYTB*); two distinct haplotype groups were recorded, based on samples from northern Spain, the United Kingdom, France, northern Italy, coastal parts of Slovenia and Croatia, and Montenegro (Western Clade), as well as those from southeastern Serbia, Romania, Germany, Denmark, the Scandinavian Peninsula, and Russia (Eastern Clade). The results suggested that the postglacial recolonization of Europe would have occurred from two major lineages: one westward from Italy and one eastward from the Balkans, with a current suture zone between them localized somewhere between Germany and the Balkans.

Analysis of *R. temporaria MT-CYTB* data [14] further confirmed the existence of two distinct clades, which split some 700 thousand years (kya later in the text) ago at the onset of the Ice Age. The eastern European and Fennoscandian populations were grouped into the Eastern Clade, while the Western Clade consisted of populations from Britain, Germany, Switzerland, and Spain. Palo et al. [14] assumed that populations from eastern Europe spread from one main glacial refugium because of their genetic uniformity, while western populations might have had more ‘cryptic’ refugia in central Europe. Schmeller et al. [15] recognized the secondary contact zone of two main clades between the rivers Weser and Elbe in northern Germany. Teacher et al. [16] further contoured the western border of the Eastern Clade through northeast Spain, southeast France, northern Italy, Switzerland, and Austria, with some overlaps between two groups detected in France, Spain, and Switzerland. The authors supposed that the refugia of the Eastern Clade were probably settled in the Apennine or Balkan peninsulas, with routes of expansion directed north, into eastern Europe, and west, along the Mediterranean coast. Later, Stefani et al. [17] questioned the existence of Eastern Clade haplotypes in Italy and on the Iberian Peninsula and considered the entire Apennine Peninsula as a refugium of the Western Clade; meanwhile, Van Rensburg et al. [18] further studied genetic differentiation within the most divergent subspecies, *R. t. parvipalmata*, and found low differentiation among all analyzed individuals. This taxon was recently proclaimed as a separate species—*R. parvipalmata* [19]. The Iberian Peninsula was then indicated as the area of geographic origin of *R. temporaria* [20]. From there, the species could spread, early on, into most of Europe, where its populations persisted in multiple refugia. Later, it was concluded that *R. temporaria* recolonized western Europe almost exclusively from multiple refugia in the Italian Alps [10]. Recently, the hypothesis that the part of the secondary contact zone between two main clades occurs in Switzerland was rejected, and it was suggested that it is positioned more to the east [21].

Genetic identification of *R. temporaria* from the Balkan Peninsula has been partially carried out in these comprehensive phylogeographic studies. Despite the patchy distribution of this species in the Balkans and, therefore, its “insular” population organization [22], no unique haplotypes were detected in this geographic area ([20] and references therein). There is a huge distribution gap in the Balkan part of Serbia and in North Macedonia from North to South, i.e., along the Morava and later the Vardar river valleys, as well as along their tributaries; in the Pannonian part of Serbia (Vojvodina Autonomous Province), the European Common frog has been recorded only in the hills of the east–southeast region [23,24]. Results of previous studies suggested that the supposed suture zone between the Western and Eastern Clades in the Balkans could follow the direction of the Morava and Vardar river valleys. Therefore, our aim here is to clarify the distribution patterns of the common European frog’s main clades in the Balkan Peninsula, focusing mainly on the broad area around the Morava river disjunction. To this end, we compare the nucleotide polymorphism of two mitochondrial gene sequences (*MT-CYTB* and 16S ribosomal ribonucleic acid—*16SrRNA* later in the text) from our sample and a set of sequences imported from GenBank from a wide European distribution area. Here, we also analyze the extent of haplotype diversity within the main clades of this species in the Balkans—predominantly around supposed suture zone—compared to the rest of Europe.

## 2. Materials and Methods

We analyzed the amount of nucleotide polymorphism in two mitochondrial gene sequences—*16SrRNA* and *MT-CYTB*—which have been broadly studied in this genus [20,21]. These gene sequences are functionally different and could have experienced differential mutational rates and selective constraints during evolution. *16SrRNA* gene sequences have moderately well-conserved secondary structures among distantly related taxa [25] and are usually applied for distinguishing between well-resolved species and establishing relations between genera [26].

### 2.1. Sample Collection

**Figure 1:**
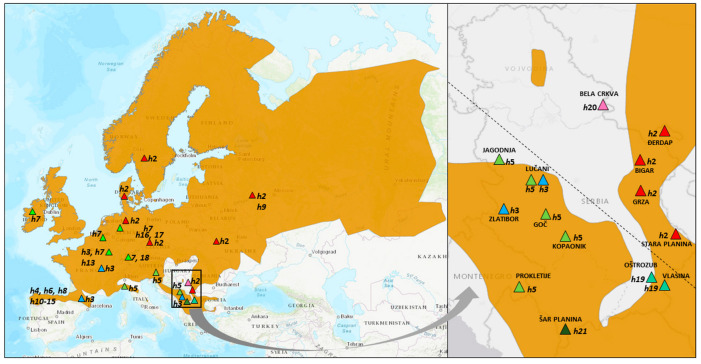
Distribution of 21 haplotypes of *R. temporaria* generated from *16SrRNA* dataset, including those imported from the GenBank. Left: European haplotypes involved in this study, with the most widespread highlighted, namely blue/green triangles *h*3, *h*5, and *h*7 (Western Clade) and red triangles *h*2 (Eastern Clade). Right: haplotypes and sampling localities in the western and central Balkans (predominantly in Serbia). “Western predominantly Serbian Clade” is represented with green/blue triangle symbols, and the “Eastern Serbian Clade” with red. (Source of the map: IUCN (International Union for Conservation of Nature) and Conservation International 2021. Rana temporaria. The IUCN Red List of Threatened Species. Version 2023-1. Accessed on 23 April 2024.).

**Figure 2:**
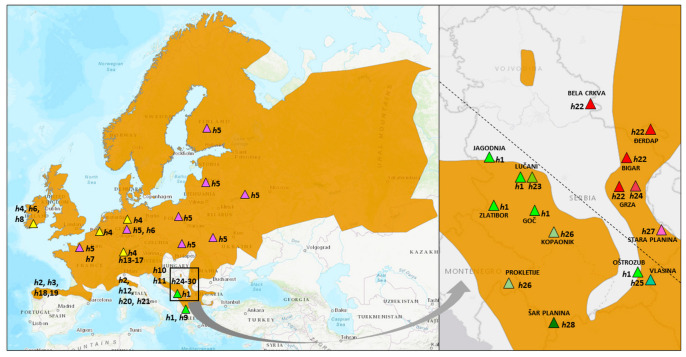
Distribution of 28 haplotypes of *R. temporaria* generated from *MT-CYTB* dataset, including those imported from the GenBank. Left: European haplotypes included in this study, with the most widespread highlighted, namely *h*4 - yellow triangles (Western Clade) and *h*5 – violet triangles (Eastern Clade). Right: haplotypes and sampling localities predominantly from Serbia, newly sampled in this study. “Western predominantly Serbian Clade” is represented with green symbols, and the “Eastern Serbian Clade” with red. (Source of the map: IUCN (International Union for Conservation of Nature) and Conservation International 2021. Rana temporaria. The IUCN Red List of Threatened Species. Version 2023-1. Accessed on 23 April 2024.).

Overall, 27 tissue samples (eggs or fingertips of adult individuals), originating from 14 different localities (Figure 1 and Figure 2), were collected within two periods: 2013–2017 (territory of the Republic of Serbia), and 1986–2007 (Batrachological collection of the Institute for Biological Research “Siniša Stanković” National Institute of the Republic of Serbia, University of Belgrade—for details, see [27]). All samples are listed in Table 1.

### 2.2. DNA Extraction, Amplification, and Sequencing

The tissue samples were preserved in 96% ethanol and were used for the extraction of total DNA with the AccuPrep Genomic DNA Extraction kit (Bioneer Corporation, Daejeon, Republic of Korea). The quantity of DNA extracts was examined by NanoPhotometer N60/N50 (Impplen, GmbH) and the quality was visualized by 1% agarose gel electrophoresis. Polymerase chain reactions (PCRs) for the amplification of *16SrRNA* mtDNA gene fragments were set using universal primers: 16Sar: 5′-CGCCTGTTTATCAAAAACAT-3′ and 16Sbr: 5′-CCGGTCTGAACTCAGATCACGT-3′ described in [6], and, for *MT-CYTB* fragments, from [20]: RCytb-F 5′-TTAGTAATAGCCACAGCTTTTGTAGGC-3′ and RCytb-R 5′-AGGGAACGAAGTTTGGAGGTGTGG-3′. The reactions were set in a GeneAmp PCR System 2700 (Applied Biosystems, Waltham, MA, USA). PCR for both genes was performed with 5x Colorless GoTaq^®^ Reaction Buffer (Promega Corporation, Madison, WI, USA), 2.5 mM MgCl2, 0.4 mM of each of the dNTPs, 0.5 µM of each amplimer, 1 U of GoTaq^®^ Hot Start DNA Polymerase (Promega), and 100 ng of genomic DNA, in a final volume of 50 µL. The temperature profiles were as described in [6] and [20] (for *16SrRNA* mtDNA and *MT-CYTB*, respectively). Since universal primers for *16SrRNA* primer were used, we paid special attention to maintaining sterile conditions, in order to avoid contamination. Sequences were provided in both directions by a third party (Macrogen, Amsterdam, The Netherlands).

### 2.3. Phylogenetic Analyses and Haplotype Diversity

All collected sequences were individually inspected using software FinchTV 1.4.0 chromatogram viewer (Geospiza Inc., Seattle, WA, USA), compared and analyzed with BioEdit Ver. 7.2.5 [28]. The occurrence of chimeric sequences and stop codons was examined and compared with available sequences in GenBank using Basic Local Alignment Search Tool (BLAST) analysis. Sequences were then aligned using ClustalW employed in MEGA Ver. X software [29,30]. Genetic diversity parameters (*h*—the number of haplotypes, *Hd*—haplotype (gene) diversity, and *Pi*—nucleotide diversity) were estimated using DNA Sequence Polymorphism Analysis of Large Datasets Version 6 - DnaSP Ver. 6 later in the text [31]. The assessment of evolutionary divergence between different sequence groups was calculated in the MEGA X software using the Kimura 2-parameter model—K2p [32]. The rate variation among sites was modelled with a gamma distribution (shape parameter = 1). Codon positions included were 1st + 2nd + 3rd + Noncoding. All ambiguous positions were removed for each sequence pair (pairwise deletion option). Evolutionary divergence rates were calculated between different predominantly “Serbian” haplotypes and, separately, between the overall European sample and the western and central Balkans sample.

Phylogeny was reconstructed for *16SrRNA* and *MT-CYTB* genes separately. For both genes, phylogenetic analysis was carried out using two different methods to confirm the strength of the tree topology: The Maximum Likelihood (ML) in MEGA X and the Bayesian analysis in MrBayes [33]. A best-fit substitution model in aligned sequences was surveyed by JModelTest v.2.1.4 [34]. The trees were created using FigTree Ver. 1.3.1 [35] (accessed on 8 August 2023) and MEGA X. The Bayesian (BI later in the text) analyses originated with random starting trees and were run for 1 × 106 generations, sampling every 100th generation, with the burn-in value set to 500. Combined trees of the various runs created a 50% majority rule consensus tree with the Bayesian posterior probability values of the relevant branches.

The haplotype network is calculated and graphically presented using Phylogenetic Network Software 10.2.0.0. (NETWORK 10.2.0.0. Software later in the text) [35] as a Median Joining Network, consisting of nodes and links (nucleotide differences), which connect the nodes. The nodes are either sequences from the dataset, or median vectors (mv)—a hypothesized, often ancestral, sequence required to connect existing sequences within the network with maximum parsimony. The evolutionary history of *16SrRNA* and *MT-CYTB* gene nucleotide sequences was inferred by using the Maximum Likelihood method and the Hasegawa–Kishino–Yano model [36] and Tamura–Nei model [37], respectively, as proposed by analysis conducted in JModelTest v.2.1.4.

## 3. Results

### 3.1. 16SrRNA Gene Nucleotide Sequence Comparison

Sequences from eight sampled tissues were submitted to the GenBank database under accession numbers PP648216–PP648224 (Table 1). The final multiple alignment of *16SrRNA* gene was 429 nt long and included 90 sequences: 8 from the field-collected samples and 82 from GenBank, including 19 sequences from our previous publication (Table S1). The total number of sites (excluding sites with gaps/missing data) was 357, and the G + C content was 0.441. There were 331 invariable (monomorphic) sites and 26 variable (polymorphic) sites, out of which 11 were found to be parsimony-informative. The overall number of *R. temporaria* haplotypes (*h*) was 21, while, in the western and central Balkans sample, *h* was 6. Their distribution is presented in Figure 1. The overall haplotype diversity (*Hd*) was 0.8689 ± 0.022, and the nucleotide diversity (per site) (*Pi*) was 0.01027 ± 0.00045.

Nucleotide diversity between six predominantly “Serbian” haplotypes (Table 2), calculated using DnaSP, demonstrated the lowest values (shown in bold), i.e., the highest similarity, between *h*5 (Jagodnja, Lučani, Goč Mt., Kopaonik Mt., and Prokletije Mt.), *h*19 (Oštrozub Mt. and Vlasina Plateau), and *h*21 (Šar Mt.) in the Western predominantly “Serbian” Clade. Similarly, haplotype *h*2 (Đerdap, Grza, Bigar, and Stara Mt.) was the most like *h*20 (Bela Crkva) in the Eastern “Serbian” Clade (Table 2). The highest nucleotide divergence was inferred between the newly discovered “Serbian” haplotypes—western *h*21 (Šar Mt.) and eastern *h*20 (Bela Crkva). Intragroup nucleotide diversity was much higher in the Western predominantly “Serbian” Clade (*Pi* = 0.00207) than in the Eastern “Serbian” Clade (*Pi* = 0.00042). Intergroup nucleotide diversity among these two clades (27 sequences) was *Pi* = 0.00791 and, of the total analyzed common European frog sample without outgroup (88 sequences), *Pi* = 0.01027.

Evolutionary analyses conducted in MEGA X involved 90 *16SrRNA* nucleotide sequences and a total of 445 positions in the final dataset. Evolutionary divergences were estimated between all 21 haplotypes generated from the total sample from Serbia and Montenegro and imported from the GenBank. Evolutionary divergence, calculated between the two clades, i.e., the Western Clade (*h*1, *h*3–8, *h*10–19, and *h*21) and the Eastern Clade (*h*2, *h*9, and *h*20), was 0.0138, and the standard error (SE later in the text) was 0.0051) (Table 3). Correspondingly, comparison between the predominantly “Serbian” haplotypes, i.e., the Western predominantly “Serbian” Clade (*h*3, *h*5, *h*19, and *h*21) and the Eastern “Serbian” Clade (*h*2 and *h*20), produced an almost identical evolutionary divergence of 0.0137 (SE 0.0056).

### 3.2. 16SrRNA Gene Phylogeny

**Figure 3:**
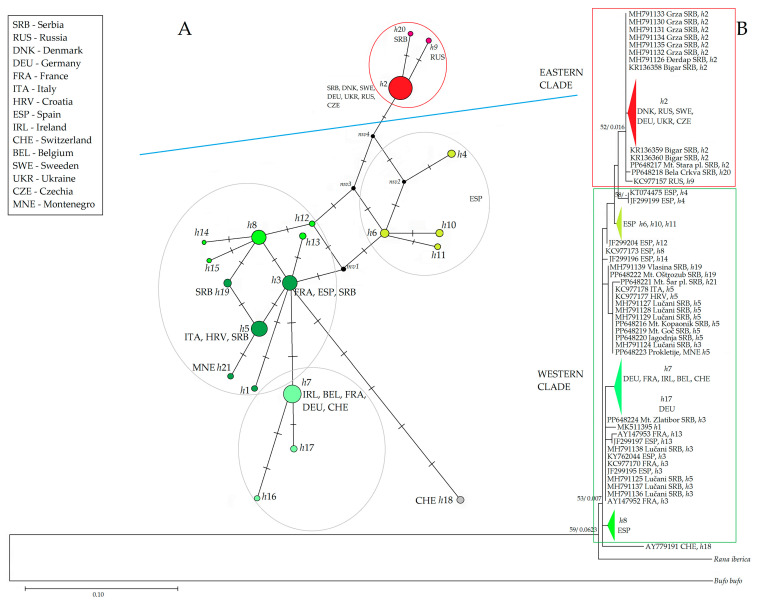
Phylogenetic relationship inferred from *16SrRNA* nucleotide comparison, presented with two trees. (**A**): Median Joining Network tree represents connections among 21 haplotypes of *R. temporaria* from *16SrRNA* dataset. Node size corresponds to a sample number of a specific haplotype; mv—a hypothesized, often ancestral, sequence required to connect existing sequences within the network with maximum parsimony. Transverse lines represent one nucleotide change. (**B**): ML phylogenetic tree. Some sample sequences belonging to the same haplotype have been condensed into haplotype groups for better visibility, and their color corresponds to the color of the nodes in the MJ network tree. Support values of both methods (ML and BI) are placed at the nodes in that order. The dash indicates branch support <50. Red square—Eastern Clade; green square—Western Clade.

Both phylogenetic methods produced trees with similar topology. Here, we present ML phylogenetic tree in Figure 3, with support values inferred from both methods. The tree was rooted by the outgroup, phylogenetically closer to *R. temporaria* than *R. iberica* and the outgroup, which was phylogenetically more distant, such as *B. bufo*. ML analysis involved 90 nucleotide sequences and there was a total of 445 positions in the final dataset. The tree with the highest log likelihood (−1258.02) is shown. The evolutionary history was inferred by using the Maximum Likelihood method and the Hasegawa–Kishino–Yano model [36]. A discrete Gamma distribution was used to model the evolutionary rate differences among sites (five categories (+G, parameter = 0.7515)). The rate variation model allowed for some sites to be evolutionarily invariable ([+I], 40.27% sites). The tree is drawn to scale, with branch lengths measured in the number of substitutions per site.

At the base of the ML tree is the Western Clade—a heterogeneous group of haplotypes containing fourteen different haplotypes, four of which were found in the central and western Balkans (Serbia and Montenegro). Most haplotypes of the Western Clade were detected within sample group from Spain. They were divided into two substantially diverged clades: the first was gathered around haplotypes *h*3 and *h*8 and the second around haplotype *h*6. The haplotype *h*3 was widespread: in addition to Serbia, it was also found in France and Spain. We found it in Serbia at two sampling sites, Zlatibor Mt. and Lučani. This haplotype had only one nucleotide change, related to *h*5, which was prevalent in western, southwestern, and central Serbia, and northeastern Montenegro (Jagodnja, Lučani, Goč Mt., Kopaonik Mt., and Prokletije Mt.). The haplotype *h*21 from the Šar Mt. recently separated from *h*5. In addition to the central and western Balkans, *h*5 was also detected in Croatia and Italy. The most distant subclade was the one containing haplotype *h*7, which was most common in western Europe (Ireland, Switzerland, Germany, France, and Belgium). The haplotypes *h*16 and *h*17, which were detected in Germany, were directly derived from *h*7. There was also haplotype *h*18, from Switzerland, that deviated most strongly from the Western group (five nucleotide changes).

The Eastern Clade comprised only three haplotypes, two of which were found in Serbia. The haplotype *h*2 from the first subclade was detected in the largest geographical area: from Denmark and Sweden in the northern European region, across Russia, Ukraine, and the Czech Republic in the east, to the central Balkans in the south. In Serbia, it was found in four localities in the eastern/southeastern part of the country: Đerdap, Grza, Bigar, and Stara Mt. A sample from Bela Crkva in southeastern Vojvodina (*h*20) differed from *h*2 by only one nucleotide change (same as *h*9 in Russia). It was placed outside the distributional range reported in the IUCN distributional map for *R. temporaria*. This haplotype, as well as *h*6, found in the Spanish subclade, were linked to haplotypes of the Western Clade by hypothetical evolutionary intermediaries, i.e., median vectors.

### 3.3. MT-CYTB Gene Nucleotide Sequence Comparison

*MT-CYTB* sequences from sampled tissues were submitted to the GenBank database under accession numbers PP695247–PP695273 (Table 1). The final *MT-CYTB* gene multiple alignment comprised 114 sequences: 27 from our field-collected samples and 87 from GenBank (Table S2). The total number of sites (excluding sites with gaps/missing data) was 273, and the G + C content was 0.423. There were 248 invariable (monomorphic) sites and 25 variable (polymorphic) sites, out of which 17 were found to be parsimony-informative. The total number of mutations was 27. The overall number of common European frog haplotypes (*h*) was 28, and, among samples from the western and central Balkans, *h* = 8. Their distribution is presented in Figure 2. The overall haplotype diversity, *Hd*, was 0.903 ± 0.017; nucleotide diversity (per site), *Pi*, was 0.01781 ± 0.00140.

Nucleotide diversity (*Pi*) among eight predominantly “Serbian” haplotypes (Table 4) demonstrated the highest similarity (lowest values) between *h*1 (Jagodnja, Zlatibor Mt., Goč Mt., and Oštrozub Mt.), *h*23 (Lučani), *h*25 (Vlasina Plateau), *h*26 (Kopaonik Mt. and Prokletije Mt.), and *h*28 (Šar Mt.) in the Western predominantly “Serbian” group, as well as between *h*22 (Grza, Đerdap, Bela Crkva, and Bigar), *h*24 (Grza), and *h*27 (Stara Mt.) in the Eastern “Serbian” group. The highest *Pi* was calculated between *h*25 (Vlasina Plateau) and *h*27 (Stara Mt.). Intragroup nucleotide diversity for the Western predominantly “Serbian” Clade and Eastern Serbian Clade was 0.00223 and 0.00197, respectively, while, between those two groups, it was 0.01505 (27 sequences).

Evolutionary analyses conducted in MEGA X involved 114 *MT-CYTB* nucleotide sequences and a total of 569 positions in the final dataset. Evolutionary divergences were estimated between all 28 haplotypes and between two groups: the Western and Eastern Clade. Evolutionary divergence between the Western Clade (*h*1–*h*4, h*8*–*h*21, *h*23, *h*25, *h*26, and *h*28) and Eastern Clade (*h*5, *h*22, *h*24, and *h*27) was 0.0282 (SE 0.0083). In comparison to this value, two groups of predominantly “Serbian” haplotypes—Western predominantly “Serbian” group (*h*1, *h*23, *h*25, *h*26, and *h*28) and Eastern “Serbian” group (*h*22, *h*24, and *h*27)—showed a higher evolutionary divergence of 0.0344 (SE 0.0082) (Table 5).

### 3.4. MT-CYTB Gene Phylogeny

**Figure 4:**
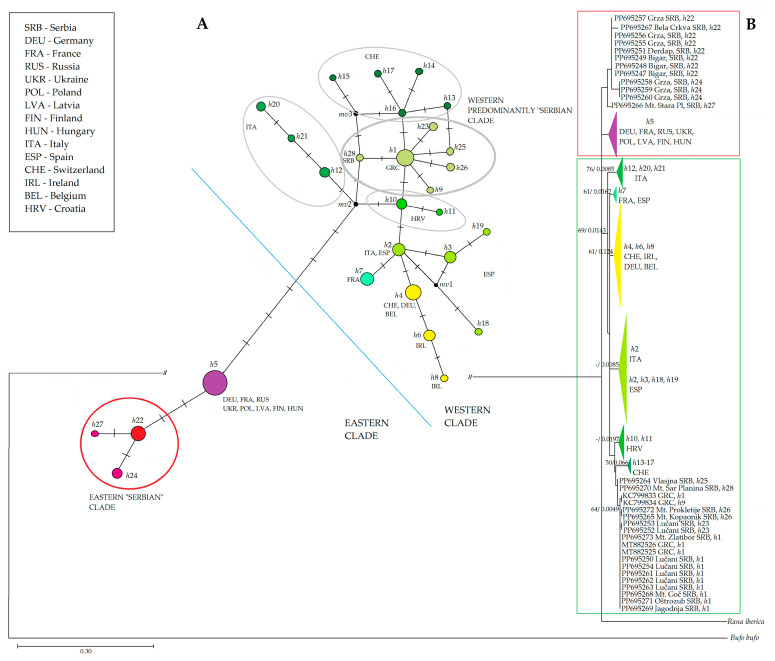
Phylogenetic trees inferred from *MT-CYTB* nucleotide comparison. (**A**): Median Network tree generated using NETWORK software represents connections among all 28 haplotypes from *MT-CYTB* dataset. Node size corresponds to a sample number of a specific haplotype; mv—a hypothesized, often ancestral, sequence required to connect existing sequences within the network with maximum parsimony. Transverse lines represent one nucleotide change. (**B**): ML phylogenetic tree with bootstrap values presented beside the nodes. Some sample sequences belonging to the same haplotype have been condensed into haplotype groups for better visibility and their color corresponds to the color of the nodes in the MJ network tree. Support values of both methods (ML and BI) are placed at the nodes in that order. The dash indicates branch support <50. Red square—Eastern Clade; green square—Western Clade.

Both phylogenetic methods produced trees with similar topologies. Here, we present ML phylogenetic tree in Figure 4. ML analysis involved 119 nucleotide sequences, and there was a total of 569 positions in the final dataset. The tree with the highest log likelihood (−1951.63) is shown. The percentage of trees in which the associated taxa clustered together is shown next to the branches. Initial tree(s) for the heuristic search were obtained automatically by applying Neighbour-Join and BioNJ algorithms to a matrix of estimated pairwise distances.

The evolutionary history was inferred on a dataset that involved 114 nucleotide sequences and a total of 569 positions, by using the Maximum Likelihood method. The tree with the highest log likelihood (−1820.16) is shown. The percentage of trees in which the associated taxa clustered together is shown next to the branches. Initial tree(s) for the heuristic search were obtained automatically by applying Neighbour-Join and BioNJ algorithms to a matrix of pairwise distances estimated using the Tamura–Nei model, and then selecting the topology with superior log likelihood value. A discrete Gamma distribution was used to model evolutionary rate differences among sites (five categories (+G, parameter = 0.9418)). The rate variation model allowed for some sites to be evolutionarily invariable ([+I], 34.89% sites). The tree is drawn to scale, with branch lengths measured in the number of substitutions per site.

As with the previous one, this tree was rooted by the outgroup—*R. iberica* and *B. bufo*. In the base of the phylogenetic tree (Figure 4) is the Western Clade, which was much more diversified, composed of 24 different haplotypes. Samples from the western and central Balkans were composed of five haplotypes: four new and one (*h*1) also detected in Greece. This clade was the most closely related to Croatian haplotypes, as well as those collected in Switzerland. Haplotype *h*4 was the most frequent in Europe from the Western Clade, found in Ireland, Switzerland, Germany, and Belgium. The Eastern Clade was composed of only four haplotypes, out of which three were found exclusively among Serbian samples. Haplotype *h*22, found in Serbia, was connected to the most widespread Eastern Clade haplotype (*h*5) by only two nucleotide changes. It extended from north-eastern Europe to certain localities in Germany and France.

## 4. Discussion

Our results represent a complement to the previous studies on *R. temporaria* phylogeography in Europe ([10,18,38] and references therein), by filling the gap in knowledge on phylogenetic relations among populations situated in the less studied area of the Balkan Peninsula. It has been suggested that postglacial recolonization of European fauna would have occurred from three major southern refugial zones [39]; in the case of *R. temporaria*, the Balkan Peninsula was supposed to be a part of the historical contact zone between Western and Eastern lineages [13]. Indeed, our results confirmed that haplotypes detected in the central Balkans belonged to both main genetic clades of the common European frog; additionally, two unique *16SrRNA* haplotypes of the Western Clade, as well as five and two unique *MT-CYTB* haplotypes of the Western and Eastern Clade, respectively, were detected in the Balkans. What was not completely known previously was the exact position of the suture zone between the two main clades in this part of the species area, and this is partially clarified in this study.

The major genetic distinction was recognized between eastern–southeastern population samples (the Eastern “Serbian” Clade) and the southeastern–southwestern—western ones (the Western predominantly “Serbian” Clade). The border zone between the two was contoured by the Morava/southern Morava river valley, towards the confluence of the Nišava river, and then by the Nišava river valley to the east. Distribution of haplotypes and their relatedness reflect historical routes connecting populations at the western edge of the Rhodope (or Serbian–Macedonian) Massif (for the explanation see [40] and references therein) to those distributed throughout the Scardo-Pindus and Dinaric mountain ranges on the southwest and west of the central Balkans. All these populations belong to the Western Clade. The fact that populations situated in the westernmost part of the Stara (Balkan) Mountains are nested within the Eastern Clade indicates that some historical barrier separated common European frogs of the Balkan Mountain Belt from those inhabiting geographically close mountains around Vlasina Plateau (Rhodope/Serbian–Macedonian Massif). The split of two main lineages could have happened as far back as between 0.215 Mya and 0.71 Mya, as proposed by [18]. These authors supposed that separation of the two main clades may have occurred much earlier than estimated.

The distribution pattern of the two major haplotype groups within *R. temporaria* remind one, on thinking of spatial genetic structuring, of viviparous populations of another boreal zoogeographic element—the European common lizard *Zootoca vivipara* [41]. Viviparity in this species evolved during/after the Pleistocene glaciations and rapidly spread among the southeastern populations, somewhere between the Balkan Peninsula and the south of Russia [42]. It is supposed that newly evolved viviparous populations would have (re)colonized northeastern and northwestern countries during interglacial periods (including the Holocene) [43]. Recently, Horreo et al. [44] have shown that (similar to the phylogeographic substructuring of the common European frog), haplotypes of European common lizard populations from eastern Europe (Russia, Belarus, Poland, Ukraine, and Romania), the Baltic countries, Finland, and neighboring parts of Sweden cluster together; the remaining northern, western, and central European populations cluster with those of the western (Montenegro, central Serbia) and central Balkans (Pirin Mt. in western Bulgaria). The authors supposed that dry Mediterranean biota extended in lowlands during the warmer postglacial period of the late Pleistocene, which could have become barriers to the spreading of elements of boreal fauna, including *R. temporaria*, in certain directions from certain refugia [44].

The presence of unique haplotypes and the structuring of the Western Clade in the area of the central and western Balkan Peninsula suggests the existence of more than two smaller refugia for the common European frog within this large southeastern European refugium. It further supports the view that the influence of different processes shaped the phylogeography of this species in a quite discontinuous landscape and “across a diverse topography” [38]. The distribution of haplotypes presented in this study showed that populations of the Western Clade comprised a higher overall number of unique haplotypes than those belonging to the Eastern Clade. Zeissett and Beebee [45] previously offered an explanation that there was only one cryptic refugium for *R. temporaria* in the climatically more monotonous eastern part of the continent, in comparison to multiple ones in western Europe. Our study suggests that multiple refugia could also have existed in the western and central Balkans (within the distribution areas of both main clades). However, the possible impact of insufficient sampling on the obtained lower haplotype diversity within the Eastern Clade could not be neglected, as this study detected new haplotypes in the previously unsampled areas of northeastern, eastern, and southeastern Serbia. The detection of additional haplotypes on the Carpathian–Balkan mountain belts could happen in the future, which would contribute to the increase in overall haplotype diversity in the Eastern Clade. Unique haplotypes discovered in the southernmost part of the distribution area of the mainly homogeneous Eastern Clade are important findings because they reflect dynamic geological and/or climatic history of that part of the central Balkans.

In comparison with the overall haplotype diversity of *R. temporaria* in Europe, a relatively high diversity of the two analyzed mtDNA sequences, especially of *MT-CYTB* sequence, was detected in the limited territory of the Balkan Peninsula. The detected level of evolutionary divergence in these two gene sequences was similar between the Western predominantly “Serbian” Clade and the Eastern “Serbian” Clade, in comparison to those recorded between Eastern and Western Clades of the entire sample analyzed here. The Pleistocene glaciations were relatively recent historical events, which caused the establishment of many temporary refugial areas in Europe [39]. The difference between the Balkan Peninsula and the rest of Europe, regarding the comparison of the extent of evolutionary divergence between the two main clades of *R. temporaria*, is quite interesting when a fast-evolving gene sequence (i.e., *MT-CYTB*) is analyzed: the higher haplotype divergence recorded in the southeast of Europe, in comparison to its central and western parts, indicates the longer and stronger isolation of local populations.

The Balkan Peninsula undoubtedly was an important Pleistocene refugium for European herpetofauna; for a number of analyzed species, populations from the Balkans are genetically closer to western than eastern conspecifics, thus reflecting the possible direction of recolonization routes [46]. Regarding the common European frog, this study revealed a relatively high number of unique haplotypes in populations distributed in the western and central Balkans. These results suggest that the Balkan Peninsula could be qualified as another “hot spot” on the map of haplotype richness of this species. Recognizing and mapping rare and/or unique haplotypes, as well as applying appropriate conservation measures in a timely manner is of utmost importance for the conservation of the species’ genetic diversity in the area of concern. For that purpose, more samples per locality are needed for the appropriate approximation of the population’s genetic structure using microsatellite analysis, which is planned to be undertaken in the future.

## 5. Conclusions

The Balkan Peninsula was an important Pleistocene refugium for the common European frog. This study recognized that one part of the suture zone between the two main genetic clades in Europe occurs in the central part of the Balkans. Moreover, populations from the western and central Balkans are characterized by a relatively high number of unique haplotypes. Recognizing and mapping rare and/or unique haplotypes, as well as applying appropriate conservation measures in a timely manner is of utmost importance for the conservation of the species’ genetic diversity in the area of concern.

## Figures and Tables

**Table 1:** List of samples with names of geographic localities and information on type of sequence analyzed with the GenBank accession numbers and a haplotype. (SRB—Serbia; MNE—Montenegro).

	Laboratory No.	*16SrRNA* GenBank No./Haplotype	*MT-CYTB* GenBank No./Haplotype	Sampling Locality	Coordinates
1	RT_3_M_RS	/	PP695247/*h*22	Bigar, SRB	44.2238, 21.8722
2	RT_6_M_RS	/	PP695248/*h*22	Bigar, SRB	44.2238, 21.8722
3	RT_7_M_RS	/	PP695249/*h*22	Bigar, SRB	44.2238, 21.8722
4	RT_1-20_RS	/	PP695250/*h*1	Lučani, SRB	43.8600, 20.1689
5	RT_M14_RS	/	PP695251/*h*22	Đerdap, SRB	44.3935, 22.1743
6	RT_M28_RS	/	PP695252/*h*23	Lučani, SRB	43.8600, 20.1689
7	RT_M29_RS	/	PP695253/*h*23	Lučani, SRB	43.8600, 20.1689
8	RT_M30_RS	/	PP695254/*h*1	Lučani, SRB	43.8600, 20.1689
9	RT_M31_RS	/	PP695255/*h*22	Grza, SRB	44.8964, 21.6459
10	RT_M32_RS	/	PP695256/*h*22	Grza, SRB	44.8964, 21.6459
11	RT_M33_RS	/	PP695257/*h*22	Grza, SRB	44.8964, 21.6459
12	RT_M43_RS	/	PP695258/*h*24	Grza, SRB	44.8964, 21.6459
13	RT_M44_RS	/	PP695259/*h*24	Grza, SRB	44.8964, 21.6459
14	RT_M45_RS	/	PP695260/*h*24	Grza, SRB	44.8964, 21.6459
15	RT_M46_RS	/	PP695261/*h*1	Lučani, SRB	43.8600, 20.1689
16	RT_M47_RS	/	PP695262/*h*1	Lučani, SRB	43.8600, 20.1689
17	RT_M48_RS	/	PP695263/*h*1	Lučani, SRB	43.8600, 20.1689
18	RT_M74_RS	/	PP695264/*h*25	Vlasina, Cvetkova reka, SRB	42.7526, 22.3263
19	RT_M91_RS	PP648216/*h*5	PP695265/*h*26	Mt. Kopaonik, Jablanova Ravan, SRB	43.2909, 20.7844
20	RT_M92_RS	PP648217/*h*2	PP695266/*h*27	Mt. Stara Planina, Kopren, SRB	43.3057, 22.8162
21	RT_M93_RS	PP648218/*h*20	PP695267/*h*22	Bela Crkva, Jaruga Stream, SRB	44.8732, 21.4127
22	RT_M94_RS	PP648219/*h*5	PP695268/*h*1	Mt. Goč, Stanišinci, SRB	43.5279, 20.8954
23	RT_M95_RS	PP648220/*h*5	PP695269/*h*1	Jagodnja, Mačkov kamen, SRB	44.3299, 19.2926
24	RT_M96_RS	PP648221/*h*21	PP695270/*h*28	Mt. Šar planina, SRB	42.0957, 20.8257
25	RT_M97_RS	PP648222/*h*19	PP695271/*h*1	Mt. Oštrozub, Zeleniče, SRB	42.8673, 22.2235
26	RT_M99_RS	PP648223/*h*5	PP695272/*h*26	Prokletije, Vusanje, MNE	42.5243, 19.8431
27	RT_M103_RS	PP648224/*h*3	PP695273/*h*1	Zlatibor, SRB	43.7003, 19.6714

**Table 2:** Nucleotide diversity (*Pi*) calculated from *16SrRNA* dataset analysis between each of six predominantly “Serbian” haplotypes (the lowest values are underlined and the highest are presented in red color).

	*h2*	*h3*	*h5*	*h19*	*h20*
*h3*	0.00598				
*h5*	0.00782	0.00124			
*h19*	0.00387	0.00222	0.00086		
*h20*	0.00083	0.00466	0.00376	0.00932	
*h21*	0.00248	0.00155	0.00054	0.00311	0.01399

**Table 3:** Evolutionary divergences calculated in MEGA X, calculated from 90 *16SrRNA* nucleotide sequences including outgroup, estimated between the Western and Eastern Clades from the total European sample analyzed here, as well as Western predominantly “Serbian” and Eastern “Serbian” Clades. Standard error estimates are shown above the diagonal.

	Western Clade	Eastern Clade	Outgroup
Western Clade		0.0051	0.0288
Eastern Clade	0.0138		0.0298
Outgroup	0.2093	0.2160	
	Eastern “Serbian” Clade	Western Predominantly “Serbian” Clade	
		0.0056	
Western Predominantly “Serbian” Clade	0.0137		

**Table 4:** Nucleotide diversity calculated from *MT-CYTB* dataset analysis between each of eight predominantly “Serbian” haplotypes (the lowest values are underlined and the highest are presented in red color).

	*h*1	*h*22	*h*23	*h*24	*h*25	*h*26	*h*27
*h*22	0.01742						
*h*23	0.00105	0.01165					
*h*24	0.01252	0.00152	0.01822				
*h*25	0.00102	0.00782	0.00409	0.01420			
*h*26	0.00140	0.01325	0.00409	0.01932	0.00239		
*h*27	0.00659	0.00165	0.02045	0.00284	0.03047	0.02270	
*h*28	0.00175	0.00864	0.00682	0.01610	0.00716	0.00478	0.03405

**Table 5:** Evolutionary divergences conducted in MEGA X calculated from 119 *MT-CYTB* nucleotide sequences, estimated between the Western and Eastern Clades from the total European sample analyzed here, as well as Western predominantly “Serbian” and Eastern “Serbian” Clades. Standard error estimates are shown above the diagonal.

	Western Clade	Eastern Clade	Outgroup
Western Clade		0.0083	0.0310
Eastern Clade	0.0282		0.0300
Outgroup	0.2611	0.2481	
	Eastern “Serbian” Clade	Western Predominantly “Serbian” Clade	
		0.0082	
Western Predominantly “Serbian” Clade	0.0344		

## Data Availability

DNA nucleotide sequences were deposited in the NCBI GenBank, and can be assessed at https://www.ncbi.nlm.nih.gov/genbank, accessed on 5 March 2024.

## Supplementary Materials

The following supporting information can be downloaded at: https://www.mdpi.com/article/10.3390/ani14101430/s1. Table S1: imported sequences of *Rana temporaria 16SrRNA* gene fragment; Table S2: imported sequences of *Rana temporaria MT-CYTB* gene fragment.
