# Supplementary material for: Phylogeographic Substructuring in the Southernmost Refugium of the European Common Frog Rana temporaria
Source: Animals (Basel). 2024 May 10;14(10):1430. doi: 10.3390/ani14101430 (PMC11117212; doi:10.3390/ani14101430)
Supplement: Supplementary file 1 [file animals-14-01430-s001.zip › animals-2931490-supplementary.pdf]

Table S1. A list of samples imported from GenBank, with IDs (GenBank accession numbers) for *16SrRNA* gene sequences.

	GenBank ID	Country of origin	Haplotype
1.	KR136358	Serbia	*h*2
2.	KR136359	Serbia	*h*2
3.	KR136360	Serbia	*h*2
4.	MH791124	Serbia	*h*3
5.	MH791125	Serbia	*h*5
6.	MH791126	Serbia	*h*2
7.	MH791127	Serbia	*h*5
8.	MH791128	Serbia	*h*5
9.	MH791129	Serbia	*h*5
10.	MH791130	Serbia	*h*2
11.	MH791131	Serbia	*h*2
12.	MH791132	Serbia	*h*2
13.	MH791133	Serbia	*h*2
14.	MH791134	Serbia	*h*2
15.	MH791135	Serbia	*h*2
16.	MH791136	Serbia	*h*3
17.	MH791137	Serbia	*h*3
18.	MH791138	Serbia	*h*3
19.	MH791139	Serbia	*h*19
20.	MK013973	no data	*h*2
21.	MH105087	Denmark	*h*2
22.	MH105086	Denmark	*h*2
23.	LC640540	Russia	*h*2
24.	KJ128957	Sweeden	*h*2
25.	KJ128956	Sweeden	*h*2
26.	KJ128955	Sweeden	*h*2
27.	KC977165	Germany	*h*2
28.	KC977163	Germany	*h*2
29.	KC977162	Germany	*h*2
30.	KC977158	Ukraine	*h*2
31.	AB058882	Russia	*h*2
32.	AB685766	Czechoslovakia	*h*2
33.	KX269196	Ukraine	*h*2
34.	KY762044	Spain	*h*3
35.	KC977170	France	*h*3
36.	JF299195	Spain	*h*3
37.	AY147952	France	*h*3
38.	MK511395	no data	*h*1
39.	KT074475	Spain	*h*4
40.	JF299199	Spain	*h*4
41.	KC977178	Italy	*h*5
42.	KC977177	Croatia	*h*5
43.	KC977176	Spain	*h*6
44.	KC977175	Spain	*h*6
45.	JF299203	Spain	*h*6
46.	KC977174	Switzerland	*h*7
47.	KC977166	Ireland	*h*7
48.	KC977167	Ireland	*h*7
49.	KC977164	Germany	*h*7
50.	KC977161	Germany	*h*7
51.	KC977160	Germany	*h*7
52.	KC977159	Germany	*h*7

53.	JF299201	Germany	*h*7
54.	AF249048	Belgium	*h*7
55.	AY147954	France	*h*7
56.	AY326063	no data	*h*7
57.	DQ283129	Germany	*h*7
58.	DQ283128	Ireland	*h*7
59.	KC977173	Spain	*h*8
60.	JF299194	Spain	*h*8
61.	AF275742	Spain	*h*8
62.	AF275741	Spain	*h*8
63.	AF275740	Spain	*h*8
64.	AF275739	Spain	*h*8
65.	AF275738	Spain	*h*8
66.	AF275735	Spain	*h*8
67.	AY147951	Spain	*h*8
68.	KC977157	Russia	*h*9
69.	JF299206	Spain	*h*10
70.	AY147955	Spain	*h*10
71.	JF299205	Spain	*h*11
72.	JF299204	Spain	*h*12
73.	JF299197	Spain	*h*13
74.	AY147953	France	*h*13
75.	JF299196	Spain	*h*14
76.	AF275737	Spain	*h*15
77.	AF124135	Germany	*h*16
78.	AY147956	Germany	*h*17
79.	AY779191	Switzerland	*h*18

Table S2. A list of samples imported from GenBank, with IDs (GenBank accession numbers) for *MT-CYTB* gene sequences.

	GenBank ID	Country of origin	Haplotype
1.	MT882526	Greece	*h*1
2.	MT882525	Greece	*h*1
3.	KC799833	Greece	*h*1
4.	KC800122	Spain	*h*2
5.	KC800121	Spain	*h*2
6.	KC800118	Spain	*h*2
7.	KC799859	Italy	*h*2
8.	KC799858	Italy	*h*2
9.	KT074851	Spain	*h*2
10.	KC800120	Spain	*h*3
11.	KC800119	Spain	*h*3
12.	KT074850	Spain	*h*3
13.	KT074849	Spain	*h*3
14.	KT074848	Spain	*h*3
15.	KC800108	Germany	*h*4
16.	KC800107	Germany	*h*4
17.	KC800106	Germany	*h*4
18.	KC800102	Switzerland	*h*4
19.	KC800101	Switzerland	*h*4
20.	KC800026	Ireland	*h*4
21.	KC800025	Ireland	*h*4
22.	KC800024	Ireland	*h*4
23.	KC800011	Switzerland	*h*4
24.	KC800010	Switzerland	*h*4
25.	KC800009	Switzerland	*h*4
26.	AF249078	Belgium	*h*4
27.	KC800105	Germany	*h*5
28.	KC800100	France	*h*5
29.	KC800099	France	*h*5
30.	KC800083	Rusia	*h*5
31.	KC800082	Rusia	*h*5
32.	KC800081	Rusia	*h*5
33.	KC800078	Ukraine	*h*5
34.	KC800077	Ukraine	*h*5
35.	KC800076	Ukraine	*h*5
36.	KC800055	Ukraine	*h*5
37.	KC800047	Poland	*h*5
38.	KC800046	Poland	*h*5
39.	KC800045	Poland	*h*5
40.	KC800044	Poland	*h*5
41.	KC800043	Poland	*h*5
42.	KC800019	Ukraine	*h*5
43.	KC799992	Latvia	*h*5
44.	KC799930	Rusia	*h*5
45.	KC799929	Rusia	*h*5
46.	KC799914	Finland	*h*5
47.	KC799913	Finland	*h*5
48.	KC799912	Finland	*h*5
49.	KC799911	Finland	*h*5
50.	KC799910	Finland	*h*5
51.	KC799909	Finland	*h*5

52.	KC799908	Finland	*h*5
53.	KC799850	Hungary	*h*5
54.	KC799823	Ukraine	*h*5
55.	KC800104	Germany	*h*6
56.	KC800022	Ireland	*h*6
57.	KC800021	Ireland	*h*6
58.	KC800020	Ireland	*h*6
59.	KC800104	Germany	*h*6
60.	KC800098	France	*h*7
61.	KC800097	France	*h*7
62.	KC800096	France	*h*7
63.	KC800095	France	*h*7
64.	KC800094	France	*h*7
65.	KC800080	France	*h*7
66.	KC800023	Ireland	*h*8
67.	KC799834	Greece	*h*9
68.	KC799822	Croatia	*h*10
69.	KC799821	Croatia	*h*10
70.	KC799820	Croatia	*h*10
71.	KC799819	Croatia	*h*10
72.	KC799817	Croatia	*h*10
73.	KC799818	Croatia	*h*11
74.	KC799801	Italy	*h*12
75.	KC799800	Italy	*h*12
76.	KC799799	Italy	*h*12
77.	MF624355	Switzerland	*h*13
78.	MF624354	Switzerland	*h*14
79.	MF624353	Switzerland	*h*15
80.	MF624352	Switzerland	*h*16
81.	MF624351	Switzerland	*h*17
82.	KY762154	Spain	*h*18
83.	KT074852	Spain	*h*19
84.	KT074790	Italy	*h*20
85.	KT074788	Italy	*h*20
86.	KT074789	Italy	*h*21
